# Applying a simplified economic evaluation approach to evaluate infertility treatments in clinical practice

**DOI:** 10.1093/humrep/dead265

**Published:** 2023-12-26

**Authors:** Qian Feng, Wentao Li, Emily J Callander, Rui Wang, Ben W Mol

**Affiliations:** Department of Obstetrics and Gynaecology, Monash University, Clayton, VIC, Australia; Department of Obstetrics and Gynaecology, Monash University, Clayton, VIC, Australia; Discipline for Health Services Management, School of Public Health, University of Technology Sydney, Sydney, NSW, Australia; Department of Obstetrics and Gynaecology, Monash University, Clayton, VIC, Australia; Department of Obstetrics and Gynaecology, Monash University, Clayton, VIC, Australia; Aberdeen Centre for Women's Health Research, School of Medicine, University of Aberdeen, Aberdeen, UK

**Keywords:** IVF, infertility treatments, cost effectiveness, add-ons, benchmark for cost-effectiveness

## Abstract

IVF is the backbone of infertility treatment, but due to its costs, it is not affordable for everyone. The cost of IVF is further escalated by interventions added to the routine treatment, which are claimed to boost pregnancy rates, so-called add-ons. Consequently, it is critical to offset the increased costs of an intervention against a potentially higher benefit. Here, we propose using a simplified framework considering the cost of a standard IVF procedure to create one live-born baby as a benchmark for the cost-effectiveness of other fertility treatments, add-ons inclusive. This framework is a simplified approach to a formal economic evaluation, enabling a rapid assessment of cost effectiveness in clinical settings. For a 30-year-old woman, assuming a 44.6% cumulative live birth rate and a cost of $12 000 per complete cycle, the cost to create one live-born baby would be ∼$27 000 (i.e. willingness to pay). Under this concept, the decision whether to accept or reject a new treatment depends from an economic perspective on the incremental cost per additional live birth from the new treatment/add-on, with the $27 000 per live-born baby as a reference threshold. This threshold can vary with women’s age, and other factors such as the economic perspective and risk of side effects can play a role. If a new add-on or treatment costs >$27 000 per live birth, it might be more rational to invest in a new IVF cycle rather than spending on the add-on. With the increasing number of novel technologies in IVF and the lack of a rapid approach to evaluate their cost-effectiveness, this simplified framework will help with a more objective assessment of the cost-effectiveness of infertility treatments, including add-ons.

## Introduction

Annually, 2.6 million IVF cycles are performed globally, with >500 000 babies born via IVF each year (ESHRE, 2022; International Committee for Monitoring Assisted Reproductive Technologies, 2022). Despite this increasing magnitude of IVF, the uptake of this technology varies significantly between countries, with the proportion of infants born following IVF ranging from 9.3% of all births in Spain to 0.1% in Serbia (European IVF Monitoring Consortium (EIM), for the European Society of Human Reproduction and Embryology (ESHRE) *et al.*, 2022; Tsigdinos, 2019).

One reason driving this glaring disparity is the cost of treatment, both from a patient perspective as well as from a societal perspective in private and public systems. IVF, the cornerstone of modern fertility treatment, is not affordable for everyone due to its costs and the lack of inadequate insurance coverage (Chambers *et al.*, 2014). In the USA, the direct cost of one complete IVF cycle, which includes all fresh and frozen transfers of embryos derived from one oocyte retrieval cycle, is around US$12 000 (American Society for Reproductive Medicine, 2023; Conrad, 2023), which represents around one-fifth of the average disposable income while in many regions infertility treatment is not reimbursed (Statistia, 2022). The out-of-pocket expenditure is further escalated by a swathe of adjuvant treatments, also known as add-ons. Add-ons are variations from the routine IVF treatment that are intended to boost success rates (Harper *et al.*, 2017; Kamath *et al.*, 2019). Add-ons include, for example, endometrial receptivity assay, endometrial scratching, pre-implantation genetic screening for aneuploidy (PGT-A), and time-lapse imaging, and the list continues to grow (Kamath *et al.*, 2019). However, there is limited evidence of the effectiveness and safety of add-ons (Wilkinson *et al.*, 2019), and their economic value has received less attention.

Assessing the cost-effectiveness of the add-on use is imperative, particularly because the costs are high. The average price for time-lapse imaging is around $500 per cycle, the median cost of PGT-A is $3000 per procedure, and the cost of intravenous immunoglobulin ranges between $2000 and $14 000 per IVF cycle (van de Wiel *et al.*, 2020). Also, the cost of add-ons is often not subsidized by public or private insurance schemes, resulting in infertile couples paying significant out-of-pocket expenses. Hence, demonstrating which of these add-ons are cost-effective is critical for couples to justify the increased payment associated with the add-on use. Given that couples will generally have a finite budget from which to fund their treatment, ensuring cost-effective decision-making and maximizing health gains from a given budget is essential, in the case of IVF, to maximize the chance of a baby. As such, the cost of add-ons should be weighed against the cost of a new subsequent IVF cycle, relative to its effectiveness to ensure cost-effective use of funds.

When evaluating the value of infertility treatments, we believe that cost per live-born baby, instead of the quality-adjusted life year, is the most appropriate measurement. This is because quality-adjusted life year was initially created to capture improvements in patients’ health, not to value additional babies (Collins, 2002; Devlin and Parkin, 2003; Keller and Chambers, 2022). The main aim of infertility treatment is to help a couple or an individual in having a baby. The financial cost to achieve that live-born baby is more relevant in the comparison of fertility treatments. Incorporation of future years that a baby will live makes comparisons less insightful, while at the same time, they do not add anything to the outcome, given the fact that the life expectancies of babies born after IVF and after natural conception are, as far as we know, largely similar (Wang *et al.*, 2020).

An ESHRE Capri workshop several years ago proposed $21 000 per live birth as a reasonable cost-effectiveness ratio (ESHRE Capri Workshop, 2015). They assumed that women become pregnant within four IVF cycles and proposed rather intuitively that a total cost of $21 000 was a reasonable threshold. In a study performed in Spain a decade ago measuring the willingness to pay for women undergoing ovarian stimulation, the median of maximal willingness to pay was $800 for hormonal stimulation, and over a third of the women were willing to pay an additional $100 to $300 for a 1–2% improvement in effectiveness (Palumbo *et al.*, 2011). A figure of $200 for a 2% improvement in live birth rate converts to a willingness to pay of $10 000 for the birth of a baby. Despite its subjective nature, it provides a rapid and sensible estimate for a willingness-to-pay cost. Such benchmarks for cost-effectiveness, while not a new concept, are not systematically applied in reproductive medicine. As is standard practice in applying economics to healthcare interventions, we propose using the general cost of IVF and the general success rate of IVF as a benchmark to which the cost-effectiveness of other fertility treatments, including add-ons, can be compared. This is also known as the willingness-to-pay threshold. Below this threshold, the add-on would be considered ‘cost-effective’. As elaborated later, this threshold will vary by factors that influence effectiveness (with women’s age as the most important example), costs (for example the country where the treatment is applied or the economical evaluation perspective it was chosen), and safety issues of the treatment.

In this study, we use add-ons in IVF and interventions outside IVF as examples to demonstrate how the framework is used in reproductive medicine. Then, we discuss several considerations and caveats when using the framework. While we recognize that our proposal represents a simplified approach to a well-established framework in economic evaluation, a rapid assessment of cost-effectiveness has been rarely applied clinically in reproductive medicine. In addition, we realize that solid evidence for the effectiveness of many add-ons is still lacking and that safety data is scarce (Armstrong *et al.*, 2019; Human Fertilisation and Embryology Authority, 2019). Our intention is therefore not to promote any add-ons or to overrule the evidence in effectiveness and safety evidence, but to demonstrate the proof-of-concept of the simplified framework.

## The simplified framework explained

We start with the assumption that a single complete cycle of IVF treatment (including frozen cycles) for a woman aged 30 years has a cumulative live birth rate of 44.6% per started cycle (SART, 2020) and costs $12 000 per cycle (Patrizio *et al.*, 2022; Conrad, 2023). This implies that the cost we are roughly willing to pay is $12 000 for a chance of 44.6% of having a baby, which converts to almost $27 000 (to be precise $26 905; $12 000 divided by 44.6%) per live-born baby (see Supplementary Data File S1 for detailed calculation formulas). This $27 000 to achieve the live birth of one baby can be used as a benchmark for the willingness to pay, to which other fertility treatments and IVF add-ons can be compared. If a new therapy or add-on is claimed to improve the cumulative live birth rate compared with conventional IVF by a certain percentage, the decision of whether to abandon or accept this new treatment from an economic perspective hinges upon whether the incremental cost per additional live birth falls below the accepted cost-effectiveness threshold (i.e. the willingness to pay equals to $27 000/live birth gained) (Table 1). When the incremental cost per live birth of the new treatment falls below this threshold, adopting the add-on is considered a cost-effective use of funds, while vice versa for a cost per live birth above this threshold, the couples’ money can better be spent on a new cycle.

**Table 1.** The benchmark for cost-effectiveness based on the cost of a conventional complete IVF in relation to the success rates per age stratum (SART, 2020).

Age of women at oocyte retrieval, years	CLBR per started cycle (%)	Estimated cost per conventional complete IVF cycle	The cost to achieve a live-born baby	The maximal acceptable cost for achieving a 2% increase in CLBR
Under 35 years old	44.6	$12 000	$26 905	$538
35–37	31.5	$12 000	$38 095	$762
38–40	19.9	$12 000	$60 301	$1206
40–41	9.7	$12 000	$123 711	$2474
42 and above	2.9	$12 000	$413 793	$8276

CLBR: cumulative live birth rate.

## Use of the simplified framework to evaluate IVF add-ons

To demonstrate the simplified framework, we refer to a recent economic evaluation of endometrial scratching by van Hoogenhuijze *et al.* (2022). Their evaluation was based on the data from a multicentre randomized controlled trial (RCT) comparing endometrial scratching to no scratching before the second IVF/ICSI cycle among couples who failed their first IVF/ICSI cycle (SCRaTCH trial) (van Hoogenhuijze *et al.*, 2021). They found that, in the endometrial scratching group, the mean costs were $303 (95% CI: −$321 to $870) higher than the control group, resulting in an incremental cost-effectiveness ratio for endometrial scratching of $6219 (i.e. €5846, as per reported by the authors) per additional live birth. In their cost-effectiveness analysis, the acceptability curve showed that there is an 80% chance that endometrial scratching is cost-effective if society is willing to pay $18 600 (i.e. €17 500, as per reported by the authors) for each additional live birth. The authors concluded that they could not provide a clear-cut expenditure for one additional birth, although they allowed for estimating costs per additional live birth in different scenarios once the clinical effectiveness of scratching is known. This knowledge gap is precisely where the simplified framework could add insights. Using the simplified framework we propose, their cost of adopting endometrial scratching for one additional baby of $6219 falls below the proposed willingness-to-pay threshold to create one live-born baby at $27 000. Hence, the simplified framework informs us that endometrial scratching can be considered cost-effective under the condition that endometrial scratching increases the live birth rate by 4.8% (van Hoogenhuijze *et al.*, 2021).

A second example of applying this simplified framework is to assess whether applying physiological ICSI (PICSI) is cost-effective over ICSI alone as a fertilization method. A large RCT showed that PICSI increased the live birth rate by 2%, although the difference was not statistically significant (odds ratio in live birth rates between PICSI and ICSI: 1.12, 95% CI 0.95–1.34; *P* = 0.18) (Miller *et al.*, 2019). This 2% improvement, (if it were to be true) implies that it is acceptable to pay $540 at most (2% of $27 000, as we are prepared to pay $27 000 for one baby) for a 2% increase in the live birth rates. The $540 should then be compared to the cost of the PICSI, which is currently set at $395 (Table 2). In this case, assuming the 2% increase, for the couple PICSI would be cost-effective over ICSI alone, in order to maximize the benefit from the couple’s finite budget.

**Table 2.** The improvements in live birth rates (effectiveness) of add-ons and their costs.

Interventions (A)	Comparators (B)	Absolute CLBR differences between A and B	95% CI of the CLBR difference	Additional cost of adopting the intervention	Mean age, years (SD)	References
***Add-ons in IVF***						
PICSI	Standard ICSI	2.2%	−1.1%, 5.5%	$395	33.6 (4.4)	Hert & Essex Fertility Center (n.d.) and Miller *et al.* (2019)
Assisted hatching	No assisted hatching	1.0%	−13.2%, 15.0%	$700	34.0 (3.3)	Assisted Fertility Program (2023) and Sagoskin *et al.* (2007), and van de Wiel *et al.* (2020)
Endometrial scratching	No endometrial scratching	5.1%	−1.2%, 11.4%	$303	35.5 (range: 31.8–39.0)	van Hoogenhuijze *et al.* (2021) and van Hoogenhuijze *et al.* (2022)
Hyaluronate enriched medium	Low hyaluronate medium	8.8%	5.7%, 12.0%	$215	Median: under 35^a^	Adeniyi *et al.* (2021) and CareFertility (2023)
***Other infertility treatments***						
HSG with oil-based contrast	HSG with water contrast	10.7%	5.2%, 16.2%	$900	32.8 (IQR: 30.1–35.7)	Dreyer *et al.* (2017) and VARTA (2017)

a The exact median or mean age was not given in the original article, nor was the standard deviation.

CLBR: cumulative live birth rate; HSG: hysterosalpingography; IQR: interquartile range; PICSI: physiological ICSI.

**Figure 1.**
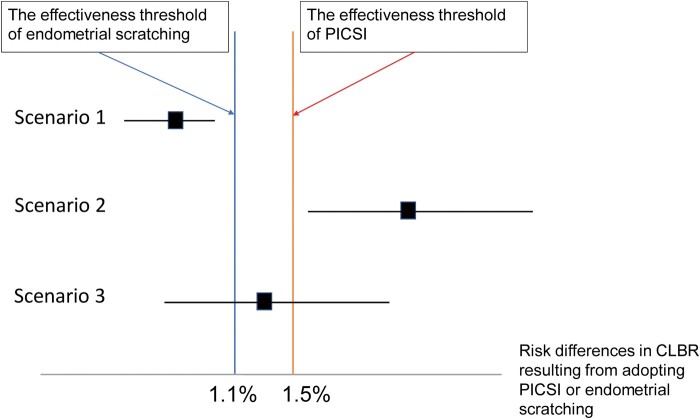
**Three scenarios where the risk difference in cumulative live birth rates obtained with adopting the tested intervention over not adopting is below, equal to, or above the effectiveness threshold.** Scenario 1: when the point estimate of the risk difference obtained with PICSI, including the upper margin of 95% CI, is lower than the threshold of 1.5%, PICSI should not be recommended. Scenario 2: A vice versa of Scenario 1. Scenario 3: when the point estimate of risk difference obtained with PICSI and its margin of 95% CI equals or crosses 1.5%, there is inadequate evidence to conclude whether adopting PICSI is cost-effective. A similar rational applies for endometrial scratching. CLBR: cumulative live birth rate; PICSI: physiological ICSI.

Alternatively, and similar to the first endometrial scratching example, we could calculate the threshold effectiveness for PICSI, which is 1.5% ($395 divided by $27 000), to assess under which scenarios the PICSI is cost-effective (Fig. 1). When the point estimate of the risk difference obtained with PICSI is lower than the threshold of 1.5%, PICSI should not be recommended. Vice versa, when the point estimate is higher than 1.5%, PICSI is cost-effective.

The 95% confidence intervals around point estimates can be used to incorporate uncertainty in the decision-making (Fig. 1). In the PICSI example, we assume (despite the lack of statistical significance) that the increase in the live birth rate for PICSI is real. We should also look at other contexts, for example, the fact that the decrease in miscarriage rate after PICSI is statistically significant, which suggests a true effect (Armstrong *et al.*, 2019; Human Fertilisation and Embryology Authority, 2019). Notably, no add-ons to date have convincingly demonstrated benefits in terms of live births, thus any of the calculations around the benchmark should consider the uncertainties regarding the estimation of absolute risk difference with careful consideration of treatment pathways and place in practice (in terms of timing of administration earlier or later in IVF treatment) (Kallogjeri *et al.*, 2020).

The simplified framework provides a benchmark cost per baby via rational and deliberate reasoning; this cost serves as a yardstick for measuring the willingness to pay derived from cost-effectiveness analyses, thus bridging the gap between the abstract willingness to pay and clear-cut practical recommendations. Truthfully, willingness to pay per se is far from a new concept, as it was produced by many cost-effectiveness analyses that help to understand the cost paid to obtain a baby (van Hoogenhuijze *et al.*, 2022). However, we are not aware that willingness to pay has been compared to the cost to create one baby as a benchmark. In the example of endometrial scratching, the authors were uncertain about the recommendation to use scratching, while their 80% chance of being cost-effective at $19 300 is below the threshold of $27 000 for cost-effectiveness as calculated by us.

## Applying the simplified framework beyond IVF add-ons

The simplified framework not only allows assessment of the cost-effectiveness of add-ons, but it can also be applied to other infertility treatments. For example, in attempting to find out whether tubal flushing with oil-based contrast medium is more cost-effective than a water-based contrast medium, large RCTs have shown that tubal flushing with oil-based contrast medium increases live births by ∼10% (Dreyer *et al.*, 2017; Zhang *et al.*, 2022). This means that it is acceptable to pay $2700 ($27 000 multiplied by 10%) at most for tubal flushing with oil-based contrast medium to obtain a 10.7% increase in the live birth rates, which is above the extra cost of using oil-based medium at $900 (van Rijswijk *et al.*, 2018). Hence, it is cost-effective to choose tubal flushing with an oil-based contrast medium over water-based ones, even at the high price of $900 but definitely at the biosimilar price of $200.

## Considerations when using the simplified framework

### Women of advanced age

The proposed benchmark is set at $27 000 per live birth. This is calculated in a scenario where the woman is 30 years old, resulting in a cumulative live birth rate of 44.6% (SART, 2020). This benchmark changes as women age due to reductions in IVF success rates. For a woman aged 40 years, the cumulative live rate plummets to 10% while the cost per IVF cycle remains almost unchanged at $12 000, driving the cost per live birth to $120 000. In this case, a 2% increase in live birth rates resulting from an add-on costs $2400. On the other hand, for women of 30 years, a 2% gain in live birth rates costs only $540, as their cost per live birth was significantly lower ($27 000) than their older counterparts (Conrad, 2023). Thus, the cost-effectiveness threshold is dependent on age and increases with increasing female age. While the effectiveness data of add-ons by women’s age is often not available, this data is not needed when using the simplified framework. Due to the reduction of success rates in IVF for women of older age, their absolute risk gained from any add-ons is lower than their younger counterparts; however, the effectiveness of the add-ons, reflected by relative risk, remains unchanged. That means the principle of the simplified framework to assess the cost-effectiveness of add-ons remains valid irrespective of women’s age. Notably, what the simplified framework suggests is not the reasonable price of add-ons but cost thresholds at which cost-effectiveness would be achieved. The simplified framework provides a transparent calculation that informs couples, insurers, and governments for decision-making of funding or reimbursing the cost-effective treatment for infertility, including add-ons.

Often, restrictions are placed on the age of women at which they are ineligible for reimbursements, implying that society is unwilling to pay a higher price for an additional live birth in women older than the cut-off age, due to the lower chance of success. In this sense, $12 000 for a chance of success of 2.9% in a woman aged 43 years corresponds to $414 000 (to be precise $413 793, $12 000 divided by 2.9%) per live birth informed by the simplified framework. This may be beyond the willingness-to-pay threshold of some societies. As there is no universally accepted cost per baby threshold for women at any age for infertility treatments, instead of ‘one price fits all’, the use of a tiered system that takes into account the actual costs associated with each age category and application of the simplified framework for information is a more thoughtful approach.

### Timing of using the add-ons

Add-ons are usually recommended after patients have encountered one or more failed cycles. The simplified framework shows however that if an add-on were found to be cost-effective, it should be offered from the first cycle onward, as the cost of one conventional IVF cycle itself is not affordable for all compared to the cost of the add-on. Hence, it would be considered irrational to hold a relatively inexpensive add-on until patients encounter failures when the add-on is more cost-effective to use earlier in treatment. It may be argued that recommending add-ons at a later time point re-ignites the hope for couples to continue the IVF journey, but this strategy does not represent the cost-effective use of finite funds and may be counterproductive. Patients may cling to the thought that they would have become pregnant already if they had been informed and had chosen the add-on in their first consultation.

### Safety issues

The safety of the mother and offspring is a factor to adjust for when using the simplified framework. When the improvements in live birth rates come at the expense of increased risk of safety, an intervention needs greater effectiveness to justify its use. For example, while heparin is claimed to potentially improve live birth rates in subfertile women undergoing IVF (Akhtar *et al.*, 2013), it also has adverse effects such as bleeding. In this scenario, the treatment effect of heparin needs to be large enough to offset possible adverse outcomes associated with heparin use (Akhtar *et al.*, 2013).

### The perspectives of the simplified framework

We take a societal perspective when explaining and using the simplified framework; changing this perspective to the couple’s or the hospital’s perspective will inevitably affect the willingness-to-pay threshold and the answer as to whether or not an intervention is cost-effective. Economic evaluation from a societal perspective should include all costs, regardless of who pays. From this perspective, the medical cost of the intervention as well as non-medical costs such as travel expenses and the costs of loss of working hours for patients should be included in the simplified framework. When taking a patient’s perspective, costs included in the simplified framework will be out-of-pocket costs for the intervention, loss of working hours and travel expenses. When clinicians apply this simplified framework to their clinical settings, they are free to choose the perspective and the corresponding cost to derive a tailored benchmark for cost-effectiveness. Likewise, when this simplified framework is used in another country where the cost of routine IVF and add-ons are different, the benchmark changes too, meaning an add-on might be cost-effective to adopt for one clinical setting but not for another. However, the principle of the simplified framework still stands, as long as the chosen perspective or the clinical setting in which the add-on is applied remains consistent throughout the evaluation.

In countries where IVF costs are covered by public funding but add-ons are not, couples may opt out of add-ons anyway because they prefer letting the insurer pay for the next IVF cycle than paying out-of-pocket for the add-ons. This scenario calls into action for insurers that if an add-on is cost-effective to add, they should consider reimbursing it to maximize the cost-effective use of funds. In other words, it is in the interest of every party involved in the infertility treatment financing, be it government or third-party subsidies, to make the adoption of cost-effective treatment actually happen.

## Conclusion

In conclusion, given that decisions in infertility treatments are intertwined with both medical and economic considerations, we propose to benchmark the cost-effectiveness of add-ons based on a willingness-to-pay threshold (e.g. $27 000 per live birth). This threshold is derived from the general cost of a complete IVF cycle and cumulative live birth rates of IVF at women’s age from a societal perspective. However, we want to point out that this threshold will change as there are cost differences between countries, as well as different perspectives. The threshold is close to the earlier threshold that was estimated rather intuitively by the ESHRE Capri Workshop (2015). The decision between spending money on the add-on or on the subsequent IVF cycle depends on whether the gains of live birth rates from an add-on are commensurate to its proportional cost per live born baby. Caveats such as the women’s age and safety issues should be heeded when using the simplified framework. With the dynamic emergence of new technologies in the arena of IVF, both patients and clinicians will be overwhelmed with more decision-making concerning additional procedures. The benchmark will continue to be a helpful tool to support such decisions.

## Supplementary Material

dead265_Supplementary_Data

## Data Availability

No new data were generated or analysed in support of this research.
