# Supplementary material for: Applying a simplified economic evaluation approach to evaluate infertility treatments in clinical practice
Source: Hum Reprod. 2023 Dec 26;39(3):448–53. doi: 10.1093/humrep/dead265 (PMC10905501; doi:10.1093/humrep/dead265)
Supplement: dead265_Supplementary_Data [file dead265_supplementary_data.pdf]

OXFORD
human reproduction

Human Reproduction, 2023, 1–1
https://doi.org/10.1093/humrep/dead265
Supplementary Data

Supplementary data file S1

The formula for how the benchmark cost per baby is calculated is as follows:

$$\text{Benchmark cost per baby} = \text{ICER} = \frac{\Delta\ C}{\Delta\ E} = \frac{C1 - \ C0}{E1 - E0}$$

$$= \frac{\$12\ 000 - \$0}{44.6\% - 0} = \$26\ 905$$

For a woman aged 30 years,

- C1 = cost for one complete IVF cycle is $12 000.
- C0 = cost for not performing IVF is 0.
- E1 = effectiveness denotes the cumulative live birth of performing one complete IVF cycle, which is 44.6%, according to the data from SART (2020).
- E0 = effectiveness of not performing IVF is 0.

Abbreviations: ICER: incremental cost-effectiveness ratio.
